# Leaf dorsoventrality as a paramount factor determining spectral performance in field-grown wheat under contrasting water regimes

**DOI:** 10.1093/jxb/ery109

**Published:** 2018-03-31

**Authors:** Omar Vergara-Díaz, Fadia Chairi, Rubén Vicente, Jose A Fernandez-Gallego, Maria Teresa Nieto-Taladriz, Nieves Aparicio, Shawn C Kefauver, José Luis Araus

**Affiliations:** 1 Integrative Crop Ecophysiology Group, Plant Physiology Section, Faculty of Biology, University of Barcelona, Diagonal, Barcelona, Spain; 2 National Institute for Agricultural and Food Research and Technology (INIA), Madrid, Spain; 3 Technological and Agricultural Institute of Castilla y León (ITACyL), Valladolid, Spain

**Keywords:** Dorsoventral effect, leaf spectroscopy, nitrogen, pigment, side-specific responses, water stress, wheat

## Abstract

The effects of leaf dorsoventrality and its interaction with environmentally induced changes in the leaf spectral response are still poorly understood, particularly for isobilateral leaves. We investigated the spectral performance of 24 genotypes of field-grown durum wheat at two locations under both rainfed and irrigated conditions. Flag leaf reflectance spectra in the VIS-NIR-SWIR (visible–near-infrared–short-wave infrared) regions were recorded in the adaxial and abaxial leaf sides and at the canopy level, while traits providing information on water status and grain yield were evaluated. Moreover, leaf anatomical parameters were measured in a subset of five genotypes. The spectral traits studied were more affected by the leaf side than by the water regime. Leaf dorsoventral differences suggested higher accessory pigment content in the abaxial leaf side, while water regime differences were related to increased chlorophyll, nitrogen, and water contents in the leaves in the irrigated treatment. These variations were associated with anatomical changes. Additionally, leaf dorsoventral differences were less in the rainfed treatment, suggesting the existence of leaf-side-specific responses at the anatomical and biochemical level. Finally, the accuracy in yield prediction was enhanced when abaxial leaf spectra were employed. We concluded that the importance of dorsoventrality in spectral traits is paramount, even in isobilateral leaves.

## Teaser

Dorsoventrality in isobilateral wheat leaves may affect the reflected radiation spectrum and its accuracy in predicting physiological and agronomical traits under field conditions

## Introduction

Spectroradiometry is a pivotal technique in the remote sensing evaluation of plant performance, used widely for precision agriculture, high-throughput phenotyping, and ecosystem studies. Very diverse information is retrieved from the spectral signature of the light reflected by the canopy or even by single leaves (Ustin *et al.*, 2009), and to date a large corpus of spectral reflectance indices has been formulated (Xue and Su, 2017). However, this information is usually predicted in an empirical manner without a clear understanding of how a basic aspect such as leaf side (adaxial versus abaxial) may affect the spectrum of the reflected radiation and the different categories of spectroradiometrical indices. This is particularly relevant because canopy evaluations of spectroradiometrical indices, at either the ground or aerial levels, are usually performed using the spectrum reflected from the adaxial part of the leaves (Jacquemoud and Baret, 1990; Sims and Gamon, 2002; Lu and Lu, 2015).

For its part, water stress is known to trigger mechanisms of acclimation in the leaf such as changes in gene expression and modification of plant physiology, morphology, and anatomy, leading to homeostatic compensation (Flexas *et al.*, 2006). At the leaf level, changes in morphology, anatomy, turgor status, and biochemical content can directly and/or indirectly impact the reflected radiation. In fact, this is the basis for plant status studies using a spectroscopic approach. Moreover, few studies have considered leaf-side-specific responses, and those focusing on the leaf dorsoventral effect have usually been performed on plant species with a clear bifacial leaf anatomy (Evans, 1999; Lu *et al.*, 2015).

In contrast, monocots such as wheat have typical isobilateral leaves whereby dorsoventral differences tend to be underestimated, considering the relative homogeneity in the mesophyll anatomy (lack of a clear dorsoventral gradient in terms of palisade versus spongy cells) and even on the epidermal surface of these leaves (which are amphystomatous). However, the abaxial epidermis is thinner and lacks bulliform cells and furrows, while the usually relatively erect position of the flag leaf and the fact that it is placed in the uppermost part of the canopy suggest that the light gradient through the leaf is not too strong.

Recent studies in monocots have reported side-specific anatomical and physiological responses that are usually dependent on genotypic and environmental effects (Soares *et al.*, 2008; Soares-Cordeiro *et al.*, 2011; Jafarian *et al.*, 2012). For instance, leaf side-specific responses of photosynthesis and stomatal closure to light intensity and CO_2_ enrichment, as well as to chilling and water stresses, have been reported in C_3_ and C_4_ species (Wang *et al.*, 1998; Soares *et al.*, 2008; Soares-Cordeiro *et al.*, 2011). Even so, there is a notable lack of understanding of how and to what extent these leaf-side-specific responses to environmental stress might affect the reflected radiation at the leaf and plant canopy scales. To the best of our knowledge, this is the first study addressing side-specific responses of wheat leaves to varying water conditions using a spectroscopic approach.

The spectral signatures in the range of visible (VIS; 400–700 nm), near-infrared (NIR; 700–1400 nm), and short-wave infrared (SWIR, 1400–2500 nm) wavelengths were investigated in the flag leaves of a set of 24 wheat genotypes growing under different water regimes in the field. Leaf reflectance spectra were recorded for the two leaf sides (adaxial and abaxial) and at the canopy level. Additionally, leaf size as well as some specific morphological and anatomical traits (epidermis, mesophyll, and xylem vessel metrics) were measured, and the stable carbon (δ^13^C) isotope composition of the flag leaves and grains as well as the total carbon (%C) and nitrogen (%N) content of the flag leaves were further analysed. Although the δ^13^C and total nitrogen content may provide information on the water and nitrogen status of the leaves (Araus *et al.*, 1997; Yousfi *et al.*, 2012), the total carbon and nitrogen and their ratio provide a broad indication of leaf chemical composition and the prevalence of structural supporting elements (Taylor *et al.*, 1989).

The aim of the present study was to investigate the effect of water regime as well as leaf-side-specific responses on leaf reflectance traits in field-grown durum wheat. Spectral reflectance indices are discussed in the context of their relationship to different physiological, biochemical, and anatomical traits of the leaf. The similarity and dissimilarities between both leaf spectra and of the spectrum of the canopy, and their implications for yield prediction were also addressed. The study was performed in the flag leaf because it has been reported to be the most isobilateral leaf in wheat (Araus *et al.*, 1986).

## Materials and methods

### Plant material and experimental set up

Field trials were carried out during the 2014/15 growing season at two locations: in central Spain at the experimental station of Colmenar de Oreja (Madrid), belonging to the Instituto Nacional de Investigación y Tecnología Agraria y Alimentaria (INIA) of Spain, and in northern Spain at the experimental station of Zamadueñas (Valladolid), belonging to the Instituto Tecnológico Agrario de Castilla y León (ITACyL). Geographic and agronomic information together with weather, irrigation, and soil information are all detailed in Table 1.

**Table 1.** Geographic, climatic, agronomic, and soil information for each study site

	Zamadueñas experimental station	Colmenar de Oreja experimental station
Altitude (m asl)	700	590
Co-ordinates	41°42'N, 4°42'W	40°04'N, 3°31'W
Mean temp.^*a*^ (°C)	10.73	13.01
Max. mea n temp.^*a*^ (°C)	17.45	21.45
Min. mean temp.^*a*^ (°C)	4.64	5.36
Precipitation^*a*^ (mm)	258.4	206.8
Sowing date	24 November 2014	21 November 2014
Harvest date	22 July 2015	20 July 2015
Sowing density (seeds m^−2^)	250	250
Plot surface (m^2^)	10.5 (7 × 1.5)	10.5 (7 × 1.5)
Irrigation provided^*b*^ (mm)	125	180
Fertilization		
First application	300 kg ha^−1^ NPK 8:15:15	400 kg ha^−1^ NPK 15:15:15
Second application	300 kg ha^−1^ CAN 27%N	150 kg ha^−1^ Urea 46%
Soil texture	Loam	Clay-loam
Soil pH	8.44	8.1

^*a*^ During the growing season.

^*b*^ In the irrigated treatments.

Twenty-four durum wheat [*Triticum turgidum* L. subsp *durum* (Desf) Husn.] varieties released during the last 40 years in Spain were grown at both experimental stations. Plots were sown in a randomized blocks design with three replicates. A total of four growing conditions were considered: rainfed and supplemental irrigated conditions for each location. At harvest, grains were dried in an oven at 60 °C for 48 h, and grain yield (GY) was determined.

### Spectral data collection and index calculation

The flag leaf spectral signature was measured with a Field-Spec4 (ASD Inc. PANalytical Company, Boulder, CO, USA) full-range portable spectroradiometer, and spectra were acquired in the 350–2500 nm range. The adaxial and abaxial leaf surfaces were measured for each leaf with an ASD leaf clip accessory assembled to an ASD standard plant contact probe coupled with a fibre optic to the FieldSpec4 spectrometer. This probe is provided with a halogen bulb and has a spot size of 10 mm diameter. A total of 288 non-senescent leaves, one per plot and thus 144 from each location, were measured at the mid grain-filling stage (25–27 May; 73 in the Zadoks scale), generating a total of 576 leaf spectra. Canopy spectra were measured at midday (80 min before and after noon) with a pistol grip coupled with the fibre optic to the FieldSpec4 spectrometer. Measurements were made 1 m above the plot canopy in a zenithal plane, and the reflectance was calibrated every 15–20 min with a Spectralon white reference panel.

A collection of 85 spectral reflectance indices (SRIs) was calculated for each leaf spectrum (Supplementary Table S1 at *JXB* online). The broadband SRIs were calculated with reference to the Landsat Enhanced Thematic Mapper Plus (ETM+) sensor (Landsat 7, USGS) wavebands.

### Other spectroradiometrically derived leaf traits

The leaf radiative transfer model, PROSPECT5 (Jacquemoud and Baret, 1990), was used to estimate the equivalent water thickness (EWT) and the total chlorophyll (Chl) and carotenoid (Car) contents from leaf reflectances using a numerical inversion of the PROSPECT 5 model via Matlab7.

Total leaf Chl content on an area basis was also assessed with a portable Chl meter (Minolta SPAD-502, Spectrum Technologies Inc., Plainfield, IL, USA).

### Leaf morphological, anatomical, and physiological traits

The leaf lamina length and width were measured in three flag leaves from each plot. Additionally, five genotypes representative of yield variability (data not shown) were selected for the anatomical observations. Flag leaf blade segments of 10 × 5 mm were sampled and submerged in Visikol clearing solution (Visikol Inc., New Brunswick, NJ, USA). The samples were incubated with 1% osmium tetroxide and 0.8% potassium ferrocyanide, washed with MiliQ water, dehydrated with acetone at 4 °C, embedded in epoxy resin at room temperature, and blocks were left in the oven for 72 h at 80 °C. Cross-sections were obtained with an Ultracut E ultramicrotome (Reichert-Jung, Vienna, Austria) and stained with methylene blue and Van Gieson’s solution. Digital images were taken with an Olympus CX41 optical microscope at ×100 and ×200 magnifications.

Images were scale-calibrated, and anatomical metrics were measured with Image J software. For the whole leaf cross-section, measurements were recorded of the leaf thickness, leaf sectional area, xylem vessel area and diameter, mesophyll cell sectional area and perimeter, epidermis cell sectional area and cell wall thickness, epidermis length, and areas of both the adaxial and the abaxial epidermis. For the cross-sections, the following ratios were calculated: total epidermis length to leaf cross-section area, the epidermis area to epidermis length ratio for the adaxial and abaxial epidermises (hereafter considered as epidermis thickness), the epidermis area to leaf area ratio for both epidermises, and the mesophyll cell area to cell perimeter ratio.

The stable carbon (^13^C:^12^C) isotope ratio as well as the nitrogen (N) and carbon (C) concentrations (%) were measured in leaf and grain dry matter using an elemental analyser (Flash 1112 EA; Thermo Finnigan, Bremen, Germany) coupled with an isotope ratio mass spectrometer (Delta C IRMS, Thermo Finnigan) operating in a continuous flow mode. Samples of 0.7–1 mg of leaf dry matter from each plot, together with reference materials, were weighed and sealed into tin capsules. Measurements were conducted at the Scientific Facilities of the University of Barcelona. Isotopic values were expressed in composition notation (δ) as follows: δ^13^C=[(^13^C/^12^C)_sample_/(^13^C/^12^C)_standard_]–1, where ‘sample’ refers to plant material and ‘standard’ to international secondary standards of known ^13^C:^12^C ratios (IAEA CH7 polyethylene foil, IAEA CH6 sucrose, and USGS 40 l-glutamic acid) calibrated against Vienna Pee Dee Belemnite calcium carbonate with an analytical precision (standard deviation) of 0.15‰.

In addition, a JEOL JSM-7100F scanning electron microscope (JEOL, Akishima, Japan) in the Scientific and Technological Facilities of the University of Barcelona was employed to observe the epicuticular wax structure of the adaxial and abaxial sides of the flag leaf. Lyophilized leaves were gold-coated and subsequently observed operating at 10 kV.

Finally, air (Testo 177-H1 Logger, Germany) and plot canopy temperature (PhotoTemp MXS, Raytek Corporation, CA, USA) were simultaneously recorded and the canopy temperature depression (CTD) was calculated as the difference between them.

### Statistical analysis

Multivariate ANOVAs were conducted using SPSS 21 (IBM SPSS Statistics 21, Inc., Chicago, IL, USA). Principal component analysis (PCA) of reflectances was performed with CANOCO 4.5 software (Ter Braak and Smilauer, 2002). Figures were drawn with SigmaPlot 10.0 (Systat Software Inc., San Jose, CA, USA). Finally, the clustered heatmap, the LASSO, and the backward stepwise regression analyses were performed with R 3.2.2 using the GPLOTS (Warnes *et al.*, 2009), the GLMNET (Friedman *et al.*, 2010), and the MASS (Ripley *et al.*, 2013) packages, respectively.

## Results

GY was significantly affected by water regime (Table 2), decreasing by 47% in Zamadueñas (from 7.17 Mg ha^−1^ to 3.77 Mg ha^−1^) and by 10% in Aranjuez (from 5.13 Mg ha^−1^ to 4.65 Mg ha^−1^) under rainfed compared with irrigated conditions. Yield differences in Aranjuez were lower due to severe lodging during grain filling in the irrigated treatment.

**Table 2.** Means and deviations of grain yield, leaf blade length and width, leaf SPAD readings, leaf nitrogen and carbon concentration (%N and %C), and its ratio (C:N), grain and leaf stable carbon isotope composition (δ^13^C), and the canopy temperature depression (CTD) for each water regime (R+, irrigated; R–, rainfed) along with the significance level of the respective one-way ANOVA

	Water regime		ANOVA
	R+	R–	*P*_WR_
Grain y ield (Mg ha^−1^)	6.95	4.81	<0.001
	0.097	0.097	
Leaf length (cm)	20.9	19.11	<0.001
	4.35	3.36	
Leaf width (cm)	1.679	1.547	0.038
	0.484	0.257	
SPAD reading	55.467	55.692	0.699
	5.189	4.621	
Plot CTD (°C)	4.89	0.63	<0.001
	1.199	1.92	
Leaf C (%)	40.13	40.42	0.486
	3.46	3.36	
Leaf N (%)	4.045	3.893	<0.001
	0.362	0.385	
Leaf C:N ratio	9.95	10.46	<0.001
	0.798	1.098	
Leaf δ^13^C (‰)	–28.1	–27.83	<0.001
	0.635	0.472	
Grain δ^13^C (‰)	–26.16	–25.34	<0.001
	0.494	1.18	

The CTD and leaf blade size (length and width) were significantly higher in irrigated conditions, whereas there was no difference in leaf Chl content assessed by a SPAD meter due to water regime. Regarding isotope composition, the δ^13^C of leaves and grains increased significantly in rainfed compared with irrigated conditions. Total leaf nitrogen content (%N) was significantly reduced in rainfed conditions, whereas the C:N ratio was increased (Table 2).

### Leaf spectrum performance

**Fig. 1.**
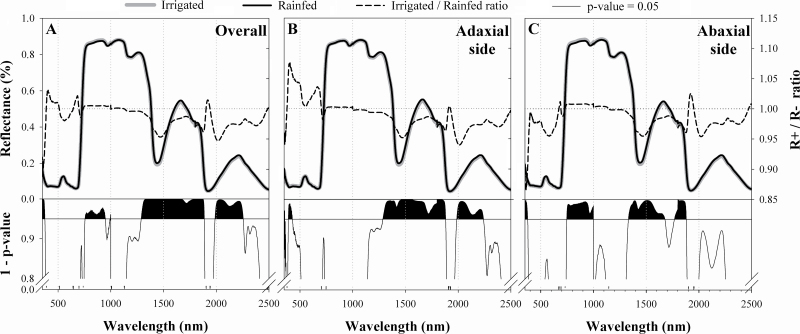
Leaf reflectance spectra in irrigated (grey line) and rainfed (black line) water conditions for the entire set of records (A, *n*=576) and separated according to the leaf side measured, either in the adaxial (B, *n*=288) or in the abaxial (C) leaf side. Below: the respective *P*-value graphs for each of the one-way comparisons performed for the reflectance throughout the spectrum, where the reflectance in each wavelength is considered as a variable and the water regime as a factor. Spectral regions where differences are significant (1–*P*-value >0.95) are shaded in black.

Water regime significantly affected leaf reflectance in the NIR and SWIR regions (Fig. 1a). Regardless of the leaf side, leaf reflectance was significantly higher under irrigated conditions in the NIR region (756–948 nm and 997–1000 nm), whereas in rainfed conditions it was higher in the violet (350–375 nm) and SWIR regions (1289–1886 nm and 1988–2254 nm).

This performance changed when studying each leaf side separately (Fig. 1b, c). Differences in NIR reflectance between water regimes were only detected in the abaxial leaf side (747–1000 nm), whereas leaves belonging to the irrigated treatment had higher reflectance. In contrast, in the second half of the SWIR region, reflectance differences between water regimes were detected only in the adaxial leaf side (1988–2240 nm), and were higher in the rainfed treatment. Finally, leaf reflectance from the end of the NIR region to the SWIR region (1300–1900 nm) was quite similar in both leaf sides, with the leaf reflectance being higher in the rainfed than in the irrigated treatment.

**Fig. 2.**
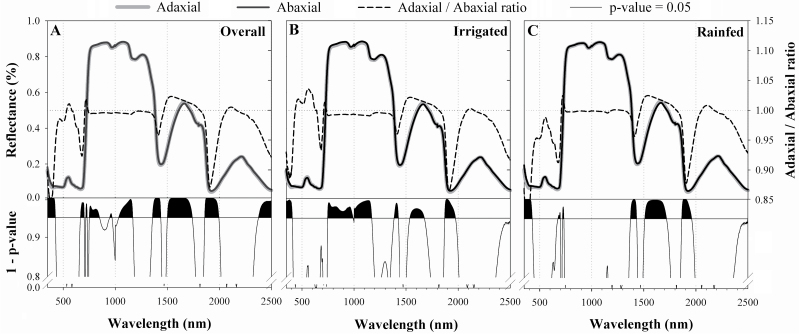
Leaf reflectance spectra in adaxial (grey line) and abaxial (black line) sides of the leaf for the entire set of records (A, *n*=576) and separating into the two water conditions, under either irrigated (B, *n*=288) or rainfed (C, *n*=288) conditions. Below: the respective *P*-value graphs for each one-way comparison performed for the reflectance across the spectrum, where the reflectance in each wavelength is considered as a variable and the leaf side as a factor. Spectral regions where differences are significant (1–*P*-value >0.95) are filled in black.

On the other hand, and regardless of the water conditions during cultivation, the reflectances of the adaxial and abaxial leaf surfaces were significantly different across several wavebands of the spectrum (Fig. 2a). In the violet region (350–425 nm), the abaxial reflectance was always significantly higher compared with the adaxial reflectance. In the red-edge region, abaxial reflectance was initially higher from 666 nm to 702 nm and then decreased significantly from 719 nm to 731 nm. Abaxial reflectance was always higher in the NIR plateau, and these differences were significant in the ranges of 752–850, 949–969, and 1035–1169 nm. Finally, in the SWIR region, adaxial reflectance was significantly higher from 1501 nm to 1739 nm and significantly lower from 1357 nm to 1437 nm, 1859 nm to 1994 nm, and 2377 nm to 2500 nm.

Additionally, differences in reflectance between leaf sides changed considerably within water regimes (Fig. 2b, c). The differences in the red-edge region between leaf sides were evident only under rainfed conditions (676–699 nm; 721–733 nm). In contrast, reflectance differences between leaf sides throughout the NIR plateau were present only under irrigated conditions (747–991 nm; 1000–1170 nm).

**Fig. 3.**
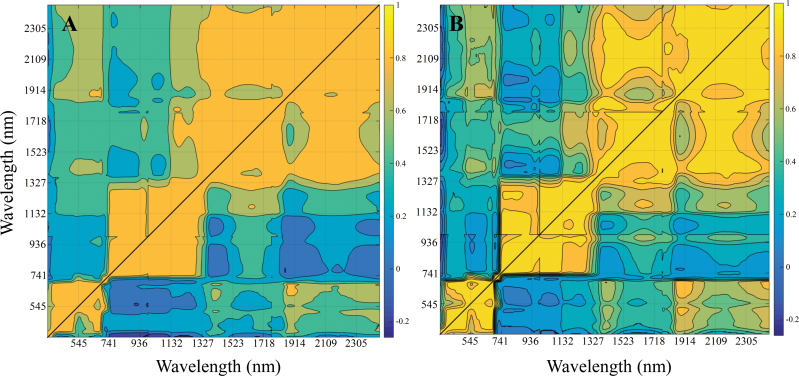
Contour maps of Pearson correlation coefficients between reflectances across the spectrum depending on water conditions (A) either in the irrigated (above the diagonal) or in the rainfed treatment (below the diagonal); and depending on leaf side (B), in the adaxial (above the diagonal) or the abaxial side of the leaf (below the diagonal).

Additionally, four contour maps were generated of reflectance cross-correlations from across the spectrum (Fig. 3) with the aim of further investigating the intrinsic relationships of the waveband reflectances for each experimental condition. The following trends can be highlighted: cross-correlation coefficients were always higher in the adaxial side of the leaf than in the abaxial leaf side (i.e. wavebands were more closely correlated in the spectrum of the adaxial leaf side). Similarly, when water regimes were compared, higher correlation coefficients were always obtained under rainfed conditions.

### Principal component analysis of reflectance ranges

**Fig. 4.**
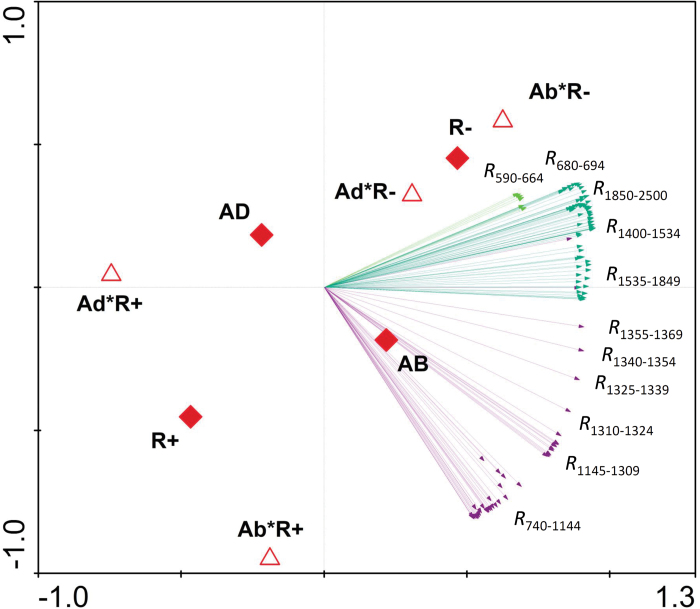
Principal component analysis of reflectances introduced as variables. Light green arrows correspond to wavebands belonging to the visible region, violet arrows correspond to wavebands in the NIR region, while dark green arrows correspond to wavebands in the SWIR region. Main levels of the factors (R+, irrigated; R–, rainfed; AD, adaxial side; AB, abaxial side) are represented as filled rhomboids, and interactions (Ad*R+; Ad*R–; Ab*R+; Ab*R–) are shown as empty triangles. The variables shown in the graph were those selected by fixing the fitness at 57%. *R*_λ_ corresponds to the reflectance at λ band.

To determine the most influential spectral ranges that accounted for data variability, a PCA was performed and reflectances in 15 nm wavelength ranges were used as variables (Fig. 4). The resulting PCA explained almost 83% of data variability (64.6% PC1 and 18.1% PC2). Variable fitness was fixed at 57%, and 123 wavebands were selected by the analysis. First, a cluster of variables grouped the wavebands in the NIR region from 740 nm to 1309 nm. This cluster was related to the leaf-side factor, and the reflectance of this region was higher in the abaxial leaf side. Secondly, the reflectance within the SWIR region, in the 1400–1534 nm and 1850–2500 nm wavebands, was moderately related to the water regime effect, with the leaf reflectance being higher under rainfed conditions. The reflectance in the VIS region (590–664 nm and 680–694 nm) had a similar trend to that of the SWIR region, but it was a milder effect. Other wavebands in the NIR and SWIR regions were also selected by the analysis, but their relationships to the studied factors were less evident. Interaction centroids in the PCA graph were very closely related in rainfed conditions (Ad*R- and Ab*R-), but not in irrigated conditions where the interaction centroids were placed far from the principal effect.

### Performance of the spectroradiometrical parameters

**Fig. 5.**
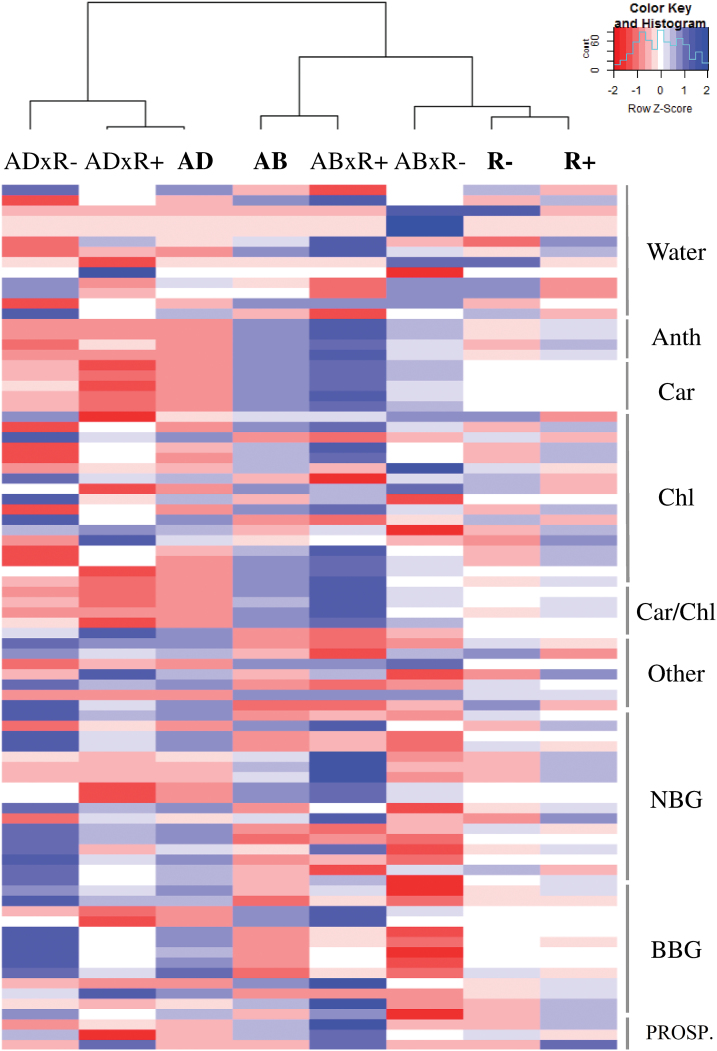
Clustered heatmap of the spectroradiometer parameters (on the right) grouped by its trait targeted as water-related SRIs (Water), anthocyanin-related SRIs (Anth), carotenoid-related SRIs (Car), chlorophyll-related SRIs (Chl), carotenoid to chlorophyll ratio-related SRIs (Car/Chl), narrowband greenness indices (NBG), broadband greenness indices (BBG); other SRIs including nitrogen- and structural-related indices and estimates from the PROSPECT model (PROSPECT). At the top, a dendrogram resulting from the clustering analysis, with labels in bold indicating the main levels of the factors (R+, irrigated; R–, rainfed; AD, adaxial side; AB, abaxial side) and the interactions (Ad*R+; Ad*R–; Ab*R+; Ab*R–). The red–blue colour scale was obtained by *Z*-score transformation of the actual values.

Factor clustering indicated that the overall differences in the spectroradiometrical parameters (including SRIs and the estimations obtained by the PROSPECT model) were greater between leaf sides than between water conditions (Fig. 5).

Water treatment differences were detected as significant by 25 of the SRIs (Supplementary Table S2), which were the most sensitive water indices, and in particular the MSI, NDII, NDWI, NMDI, and SWWI (*P*<0.001). Many other biochemical and structurally related indices (pigment, lignin, and nitrogen) such as ARI_1_, ARI_2_, NDNI, mDATT, NDLI, FRI (*P*<0.001), mCARI_2_, and MRCI (*P*<0.05) also varied significantly between water conditions. Finally, some red-edge indices including VREI_1_, VREI_2_ (*P*<0.01), and NDRE (*P*<0.05) were also shown to be sensitive to the water treatment factor.

Regarding the dorsoventral effect, 42 of the SRIs tested were sensitive to this factor. These SRIs mainly corresponded to biochemical, structural, and water indices, but also to some narrow and broadband greenness indices. The most robust differences between leaf sides were found by pigment-sensitive indices [Chl, Car, and anthocyanin (Anth)], and structural and water-related indices (*P*<0.001).

The interaction between leaf side and water regime was significant for some indices (ARI, mARI, GATB, SIPI, NPCI, SRPI, and PSRI) whose formulation included a red-edge alongside a blue or green wavelength. In such cases, the variation in these SRIs in response to water regime was clearly dependent on the side of the leaf.

Regarding the PROSPECT estimated leaf traits, only the EWT varied significantly between water regimes, and it was higher in the irrigated treatment.

### Leaf anatomy

**Fig. 6.**
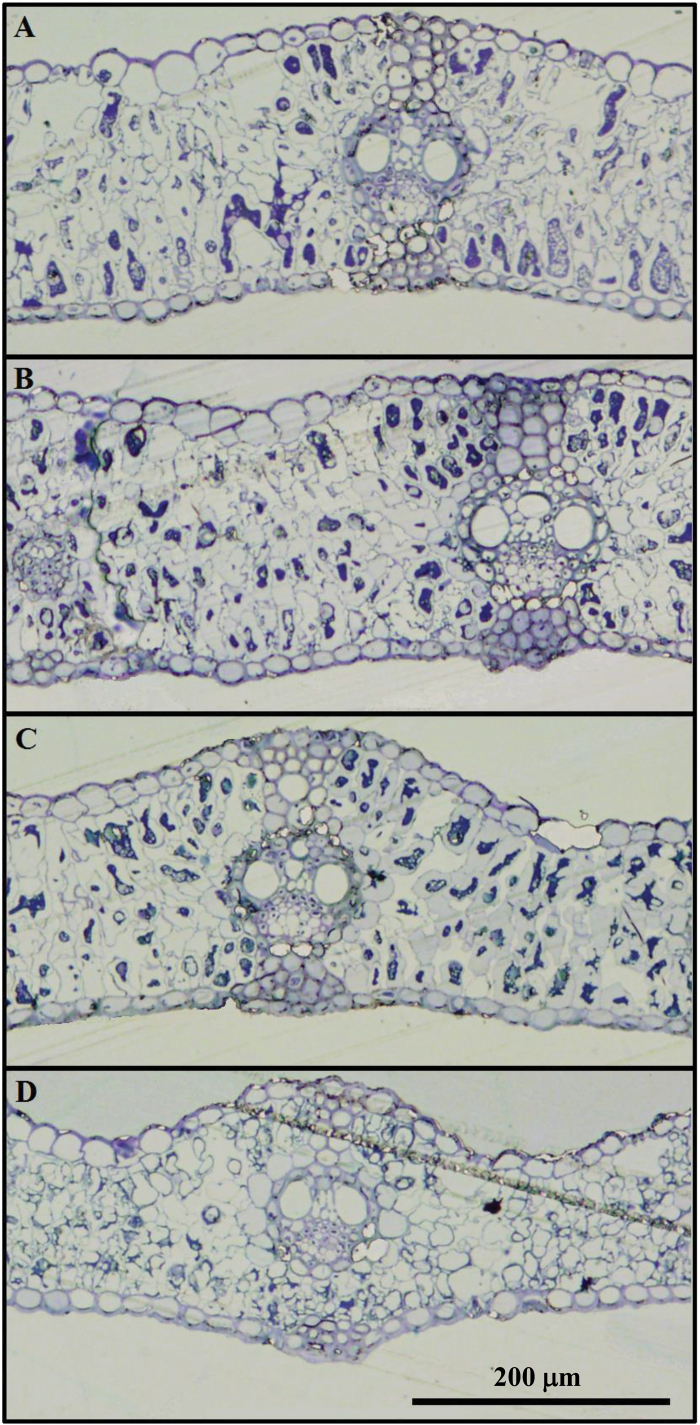
Flag leaf transverse section images of two durum wheat genotypes; (A, C) var. Tussur; (B, D) var. Avispa; grown under irrigated (A, B) and rainfed (C, D) conditions.

Leaf anatomy was studied in a subset of five genotypes with the aim of gaining insights into the relationship between leaf anatomy and spectral signature. The representativeness of the subset of samples used in the anatomical study was assessed by the respective performance of the SRIs. As in the main analysis presented here, water, nitrogen, and some pigment-related SRIs detected water regime differences for this subset of plots, while evidence for leaf side differences was mainly derived from pigment- and structure-related SRIs (Supplementary Table S3). Water stress induced significant decreases in xylem vessel cross-sectional area and diameter, and mesophyll cell cross-sectional area and perimeter, whereas the mesophyll cell cross-sectional area to perimeter ratio increased (Fig. 6; Table 3).

**Table 3.** Means of the leaf section anatomical metrics for each water regime (R+, irrigated; R–, rainfed) and leaf side along with the significance levels of the respective two-way ANOVA

	Water regime		Leaf side		Adaxial		Abaxial		Significance		
	R+	R-	Adaxial	Abaxial	R+	R-	R+	R-	P_WR_	P_LS_	P_WR*LS_
Leaf											
Thickness (µm)	199.98	176.23	-	-	-	-	-	-	.065	-	-
Perimeter/area (µm^-1^)	0.112	0.118	-	-	-	-	-	-	.350	-	-
Epidermis area/Leaf area	0.177	0.185	-	-	-	-	-	-	.249	-	-
Mesophyll			-	-	-	-	-	-		-	-
Cell area (µm^2^)	577.17	407.89	-	-	-	-	-	-	.001	-	-
Cell perimeter (µm)	111.47	88.80	-	-	-	-	-	-	.000	-	-
Cell area/Cell perimeter (µm)	5.04	4.42	-	-	-	-	-	-	.006	-	-
Xylem vessels											
Major diameter (µm)	32.50	23.28	-	-	-	-	-	-	.011	-	-
Vessels area (µm^2^)	544.34	281.69	-	-	-	-	-	-	.008	-	-
Epidermis											
Thickness (µm)	15.91	15.72	17.06	14.57	17.35	16.77	14.46	14.67	.740	.000	.477
Area/leaf area	.089	.092	.100	.081	.099	.102	.078	.083	.192	.000	.784
Cell area (µm^2^)	220.9	237.5	294.7	163.7	278.3	311.2	163.5	163.9	.385	.000	.398
Wall thickness (µm)	3.988	4.066	3.581	4.473	3.621	3.541	4.355	4.592	.643	.000	.352

All the epidermal traits measured were significantly different between leaf sides, including the area of the epidermis cell section, the epidermis thickness, and the ratio of the epidermis sectional area to the total leaf sectional area, which were higher in the adaxial epidermis, whereas the epidermis cell wall thickness was higher in the abaxial epidermis.

**Fig. 7.**
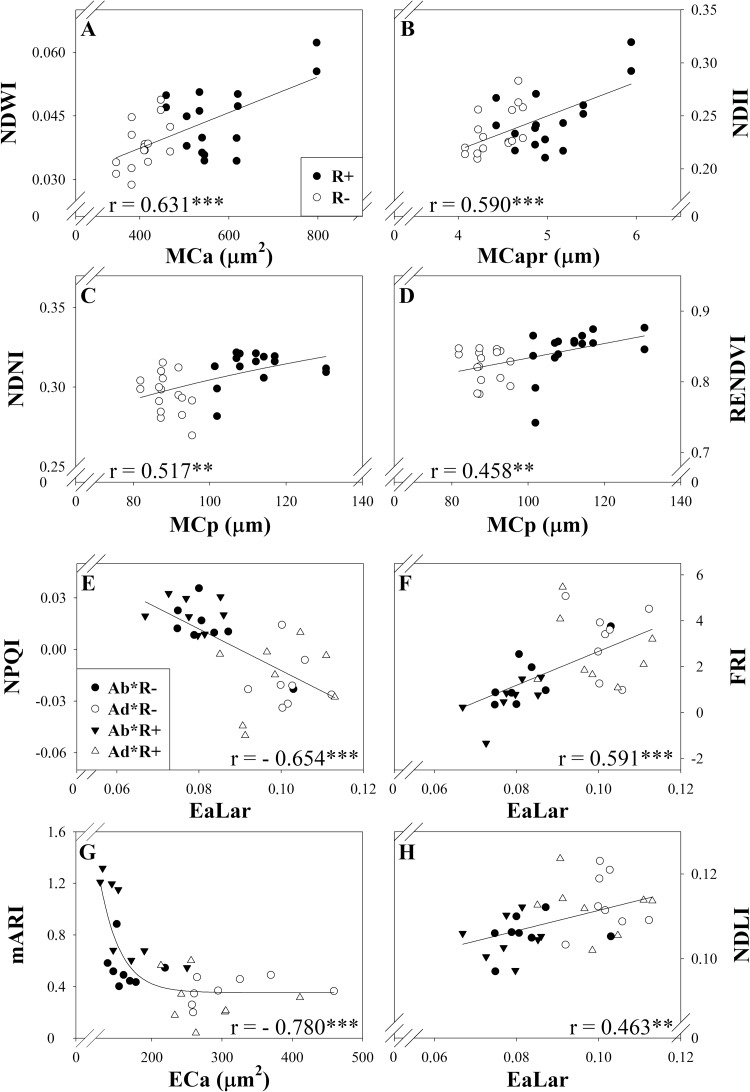
Scatter plot graphs showing correlations between anatomical leaf section traits (EaLar, epidermis sectional area to leaf sectional area ratio; ECa, epidermis cell sectional area; MCa, mesophyll cell sectional area; MCp, mesophyll cell sectional perimeter; Mcapr, mesophyll cell sectional area to perimeter ratio) and spectral reflectance indices (the water-related indices NDWI and NDII; the nitrogen-related index NDNI; the chlorophyll-related indices RENDVI and NPQI; the flavonol-related index FRI; the anthocyanin-related index mARI, and the lignin-related index NDLI) for the subset of plots selected.

Additionally, a wide range of SRIs (water-, biochemical-, and structure-related indices) significantly correlated with several anatomical traits (Fig. 7). Mesophyll cell-sectional area and the mesophyll cell area to cell perimeter ratio correlated positively with the water-related indices, NDWI and NDII, respectively (Fig. 7a, b). Meanwhile, the mesophyll cell sectional perimeter correlated positively with the N-related index, NDNI (Fig. 7c), and with the Chl-related index, RENDVI (Fig. 7d), whereas the ratio between the epidermis sectional area and the leaf sectional area correlated negatively with the Chl index, NPQI (Fig. 7e), and positively with the flavonol-related index, FRI, and with the lignin-related index, NDLI (Fig. 7f, h). Finally, the epidermis cell cross-sectional area correlated negatively with the Anth index, mARI (Fig. 6g).

### Relationship between leaf and canopy spectra and their relationship to GY

The similarities between adaxial, abaxial, and canopy reflectance spectra were assessed with a PCA, setting the reflectance spectra as variables. The resulting two-component PCA (Supplementary Fig. S1) explained 94% of the variability (82.1% PC1 and 12.7% PC2). Measurements were distributed in two parallel dot clouds, where the two leaf spectra were overlapping and parallel to that of the canopy. Leaf and canopy dot clouds were mainly separated by PC1, which was mostly dependent on *R*_1900_. In addition, from the collection of indices calculated, 74% of adaxial SRIs correlated significantly with the respective indices at the canopy level, whereas only 52% of abaxial SRIs correlated significantly with the respective canopy indices (data not shown).

In order to test the leaf-side effect on the ability for yield prediction, two multiple regression models were performed: a LASSO analysis using the full-range reflectance spectrum and a backward multiple regression using the SRIs (Table 4). In both analyses, the explained variability was always higher when using canopy spectral data, followed by the abaxial models and lastly the adaxial models, all of them being significant (*P*<0.001).

**Table 4.** Multiple regression analyses for grain yield prediction employing the adaxial, abaxial, or canopy reflectances

1^st^ Approach - Backward stepwise			
	Adaxial SRIs	Abaxial SRIs	Canopy SRIs
R^2^	0.732	0.795	0.925
adjusted R^2^	0.637	0.713	0.903
p-value	< 0.001	< 0.001	< 0.001
error of prediction	0.963	0.858	0.499
**2** ^**nd**^ **Approach - LASSO regression**			
	**Adaxial** **spectrum**	**Abaxial** **spectrum**	**Canopy** **spectrum**
R^2^ actual vs estimated	0.384	0.552	0.747
p-value	< 0.001	< 0.001	< 0.001

For the first approach, the spectral reflectance indices (SRIs) calculated at the three levels (from adaxial, abaxial, and canopy measurements) were set as variables in a backward stepwise analysis. For the second approach, three LASSO regression models were performed with the whole spectrum at the three mentioned levels. Each model was obtained using a training set (75% of data), and its robustness was assessed by its respective accuracy in predicting yield (*R*^2^) for the test set (25% of data).

## Discussion

In this study, the decrease in GY, CTD, and leaf elongation (i.e. flag leaf blade length and width) in rainfed conditions compared with the support irrigation trial was associated with water stress as shown by the increase in leaf δ^13^C and an even larger increase in grain δ^13^C (Table 2) from irrigated to rainfed conditions (Araus *et al.*, 2003; Araus *et al.* 2013; Bort *et al.*, 2014).

### Leaf anatomy-related spectral signal

A wide range of spectral traits was affected substantially by the leaf-side factor. In particular, clustering analysis (Fig. 5) revealed that the spectral parameters were more influenced by the side of the leaf measured than by the water regime.

Leaf reflectance was sensitive to leaf side in several wavebands of the spectrum (Fig. 2) that were beyond the expected structurally related wavebands (i.e. not only in the NIR but also in the red-edge and SWIR regions). Even so, PCA (Fig. 4) revealed the leaf-side factor to be strongly associated with leaf reflectance spectrum variability in the NIR region, and was positively related to the abaxial side of the leaf. The magnitude of reflectance in the NIR region is largely governed by structural discontinuities in the leaf (i.e. cell layers and interfaces) and leaf dry matter content (Peñuelas and Filella, 1998; Ceccato *et al.*, 2001; Homolova *et al.*, 2013). In particular, previous studies have reported that leaf NIR reflectance is increased in flat and thick leaves possessing thin epidermal and epicuticular layers and long palisade cells (Knapp and Carter, 1998; Johnson *et al.*, 2005; Ollinger, 2011; Kozhoridze *et al.*, 2016). In concordance, the increased NIR reflectance in the abaxial compared with the adaxial leaf side (Fig. 2) can be consistently associated with a thinner and flatter epidermis in the abaxial surface (Table 3). Thus, the existence of anatomical differences between the adaxial and abaxial leaf sides, particularly in the epidermis, greatly impacts on leaf reflectance in the NIR region, even in an isobilateral leaf.

Similarly, the reported leaf anatomical differences between water regimes, such as the larger size of the mesophyll cell cross-sectional area and perimeter and a lower cell packing level (higher cell area to perimeter ratio) in the irrigated treatment (Table 3), led to an increase in NIR reflectance (Knapp and Carter, 1998; Johnson *et al.*, 2005; Ollinger, 2011) in irrigated compared with rainfed leaves (Fig. 1). The observed trends of increasing leaf thickness and decreasing epidermis sectional area relative to the total leaf sectional area in irrigated compared with trials might also contribute to a higher NIR reflectance of the former.

PCA (Fig. 4) revealed that adaxial and abaxial reflectance properties were closely related in rainfed conditions but were markedly differentiated in irrigated conditions. In other words, water stress caused changes in the leaf that tended to reduce dorsoventral differences in the spectrum. For instance, overall reflectance differences between leaf sides were detected in the NIR region in the irrigated treatment but not in rainfed conditions (Fig. 2b, c). This trend is further supported by the contour maps of correlations between waveband reflectance for the four main effects (rainfed and irrigated conditions, adaxial and abaxial leaf sides) (Fig. 3). In these analyses, higher correlation coefficients for the adaxial side compared with the abaxial side (Fig. 3a) can be explained by lower variation (i.e. greater similarity) of the adaxial spectral traits between water regimes. Meanwhile, reflectance differences between leaf sides were lower in rainfed conditions, which explains the higher correlation coefficients in rainfed compared with irrigated conditions (Fig. 3b).

It must be noted that leaf anatomical and biochemical characteristics can be affected by the gradient of light exposure during growth. Thus, while more erect leaves are characterized by an isobilateral anatomy, more horizontal leaves may exhibit increased dorsoventrality. In the case of wheat, an insertion gradient exists, with the flag leaf exhibiting more isobilateral characteristics than the basal (tillering) leaves (Araus *et al.*, 1986), which is probably associated with a progressive increase in verticality of the successive leaves. However, environmental conditions other than the light gradient cannot be discarded. Thus an increase in the level of atmospheric drought and the intensity of solar radiation during growth seems to affect the degree of isobilaterality of the flag wheat leaf laminas growing in the field (Araus *et al.*, 1989).

In fact, as previously mentioned in this study, the leaf dorsoventral gradient was less pronounced in rainfed conditions. Nevertheless, despite there being no clear differences among the genotypes in the angle of the laminas for given growing conditions (i.e. site and water regime) (data not shown), it should be noted that during stem elongation the laminas usually extrude vertically and the final position of each lamina is only achieved when it is fully expanded.

In agreement with previous observations in cereals, the wax cover was uniform and denser on the adaxial leaf side (Supplementary Fig. S2a, c) (Araus *et al.*, 1991). As described by Willick *et al.* (2018), the filament size of the epicuticular waxes was larger and longer in the abaxial leaf side (Supplementary Fig. S2b, d), whereas water stress seemed to induce an increase in wax density (Supplementary Fig. S2c, d). Previous studies have reported that the presence of waxes (glaucousness) is important for reflecting UV–VIS light with respect to non-waxy leaves, with the UV–blue regions being particularly affected (Clark and Lister, 1975; Reicosky and Hanover, 1978; Febrero and Araus, 1994). Thus, reflectance differences between leaf sides and water regimes in the violet region could be associated with the observed structural differences in the wax cover density and size. In comparison with the waveband range of this study, changes in biochemical composition of the epicuticular waxes between leaf sides and water regimes have been reported for longer wavebands (Mid-IR) (Willick *et al.*, 2018), whereas wax determination has been addressed with shorter wavebands (UV) (Bianchi and Figini, 1986).

### Leaf water- and composition-related spectral signals

Leaf reflectance in the SWIR region has been related to water and N-protein absorption as well as to other biochemical constituents such as lignin, cellulose, and starch (Peñuelas and Filella, 1998; Ustin *et al.*, 2009; Homolova *et al.*, 2013). Even so, the amount of water available in the internal leaf structure largely controls SWIR reflectance (Ceccato *et al.*, 2001; Ollinger, 2011). Thus, the increase in leaf reflectance in the SWIR region under rainfed conditions (Fig. 1) consistently indicates lower leaf water content. At wavelengths beyond 1400 nm, water absorption partially overshadows the absorption features of other biochemical compounds (Ollinger, 2011). Nevertheless, the reported relationship between SWIR reflectance and the water regime effect (Fig. 4), as well as the performance of several SRIs, suggest that the water regime may affect leaf biochemistry in addition to the direct effect on leaf water status.

In particular, the performance of several spectral traits revealed differences in leaf N-protein and lignin content in response to water stress. The moderate relationship between the water regime effect and the second half of the SWIR region (Fig. 4) was coincident with the absorption bands of these compounds (Kokaly 2001; Ustin *et al.*, 2009). Additionally, the SRIs related to N-protein and lignin content (NDNI and NDLI, respectively) varied in response to the low water regime, showing decreasing and increasing trends, respectively (Supplementary Table S2). The analytical measurements of leaf N concentration that indicated a decrease under rainfed conditions (Table 2) further confirmed this trend. Additionally, the reported positive correlation between the mesophyll cell section perimeter and the N-related index NDNI (Serrano *et al.*, 2002) (Fig. 7c) showed the relationship between composition and anatomical changes, which were both caused by water-limited conditions.

On the other hand, increasing the leaf C:N ratio (Table 2) in rainfed conditions could indicate the prevalence of supporting elements (i.e. cell wall materials) enriched in N-free compounds such as lignin. Moreover, the described epidermal leaf side differences (Table 3; Fig. 6) (i.e. greater epidermis thickness and cell section area, and the increased percentage of epidermis area relative to the total leaf sectional area in the adaxial leaf side) and their relationship to the lignin spectral signal (Fig.7h) further support this suggestion.

Regarding the leaf water signal, the water-related SRIs derived from the SWIR bands (e.g. MSI, NDII, NMDI, SWWI, and NDryMI) were the most sensitive to changing water conditions, indicating a decrease in water content conditions as well as an increase in leaf dry matter content in response to rainfed conditions (Supplementary Table S2). This superior performance of SWIR-based SRIs has been noted previously (Carter, 1991) following the high sensitivity of reflectance to leaf water content in the water-absorbing 1300–2500 nm range. In contrast, the use of the 970 nm secondary water absorption band (which is included in indices such as WBI and NWI) was ineffective at the scale of the current work, although it has been reported as effective at the whole-plant and canopy scales (Peñuelas *et al.*, 1997; Gutierrez *et al.*, 2010). The PROSPECT EWT parameter proved to be robust, showing differences between water regimes.

In turn, for the subset of genotypes where leaf anatomy was studied, the observed variability in water-related indices, such as NDWI and NDII (Hardisky *et al.*, 1983; Gao 1996), was strongly and positively correlated with the increase in the mesophyll cell size and the decrease in the mesophyll cell packing (i.e. mesophyll cell area to perimeter ratio) from the rainfed to the irrigated treatment (Fig. 7a, b). These results show that the variation in anatomical traits (in turn caused by the water conditions during growth) affected the leaf spectral performance.

The consistent dorsoventral differences in SWIR reflectance (centred on 1600, 1400, and 1900 nm) found under both water conditions (Fig. 2) suggest the existence of constitutive differences in biochemistry and/or water content between leaf sides that are independent of the water conditions during growth. The adaxial epidermis of wheat is characterized by the presence of bubble-shaped bulliform cells whose changes in turgidity control leaf straightening. Accordingly, we hypothesized that a higher water content signal would be expected in the adaxial epidermis. The reported decrease in adaxial reflectance (Fig. 2) in the SWIR that was coincident with strong absorption by water (centred on 1430 nm and 1950 nm) may support this hypothesis as a direct response to water changes. However, the response in the NIR region has previously been reported rather as an indirect effect via changes in leaf structure and scattering (Ollinger, 2011).

Some of the water SRIs tested, particularly those using the waveband centred on 1600 nm, detected apparently higher water content in the abaxial side of the leaf. This waveband has been reported to be indirectly sensitive to leaf and canopy water content (Ceccato *et al.*, 2001; Jackson *et al.*, 2004), but it is also related to leaf structure and biochemical characteristics (Ceccato *et al.*, 2001; Serrano *et al.*, 2002). Therefore, the possible existence of biochemical and/or structural differences between the two sides of the leaf could affect the reflectance in this waveband and thus interfere with leaf water content retrieval. Nevertheless, some other water-related SRIs (NDMI_1_ and NDMI_2_) combining NIR and SWIR bands as well as the EWT were insensitive to leaf side but were effective at detecting water regime differences. Thus, combining NIR and SWIR bands may remove variations induced by mesophyll structure (Ceccato *et al.*, 2001) and avoid the dorsoventral effect by improving the accuracy of water content retrieval.

### Chlorophyll-related spectral signal

Regarding the dorsoventral effect on the Chl spectral signal, the observed significant changes in red-edge reflectance between leaf sides (Fig. 2) were largely related to absorption by Chl (Ustin *et al.*, 2009; Ollinger, 2011). Unlike other spectroscopic studies using species with bifacial leaves (Knapp *et al.*, 1988; Vogelmann and Evans, 2002; Johnson *et al.*, 2005), we detected an apparently higher Chl content in the abaxial side of the leaf from assessments with all of the Chl-sensitive spectral indices (Supplementary Table S2). The correlation between the Chl-related index, NPQI (Barnes *et al.*, 1992), and the ratio of the epidermis sectional area to the leaf sectional area (Fig. 7e) (which increased in the adaxial leaf side) revealed a dorsoventral gradient in the Chl spectral signal. The higher sun irradiance reaching the upper side of the leaf may involve a chloroplast acclimation process (i.e. lower Chl content) (Terashima *et al.*, 1986), which may support the existence of a leaf dorsoventral gradient in Chl.

Different responses of leaf Chl content to water stress have been reported in the literature (Loggini *et al.*, 1999; Carter and Knapp, 2001; Izanloo *et al.*, 2008; Valifard *et al.*, 2012), and can be highly dependent on genotypic variability and phenological stage. In this study, the total leaf Chl content per unit area (measured with a portable SPAD meter) was unaffected by water conditions. SPAD readings are exponentially correlated with leaf Chl content (Uddling *et al.*, 2007), so for high values of SPAD (as they occur in our study) the variation in the actual Chl content could be high, and the accuracy of the readings can also be affected by leaf water content (Martínez and Guiamet, 2004). Instead, many of the Chl-related SRIs (e.g. RECI, mSR_1_, mSR_2_, mDATT, VREI_1_ MRCI, and NDRE) showed interesting trends. Indices based solely on red-edge wavebands were more sensitive to the water regime and apparently indicated a decrease in Chl content under rainfed conditions, whereas SRIs including blue or green wavebands in their formulation (e.g. ChlNDI, TCI, and TCARI) were insensitive. The interference of other pigments such as Car and Anth, which have absorption wavebands in the blue and green regions, might affect the Chl retrieval of some of the SRIs tested. As additional evidence, the reflectance in the red region was shown to be positively related to rainfed conditions in the PCA (Fig. 4). Although the estimated Chl content from the PROSPECT model did not vary significantly between water conditions, it followed the same decreasing trend in rainfed conditions. Additional evidence for this is the positive correlation between the Chl-related SRI, RENDVI (Gitelson and Merzlyak, 1994), and the mesophyll cell perimeter (Fig. 7d), whereby a higher mesophyll cell size under irrigation compared with rainfed conditions matches the increase in the Chl spectral signal. Previous studies have shown that indices using off-chlorophyll absorption wavebands (i.e. 690–730 nm) are the best Chl predictors because they have greater sensitivity to subtle changes in Chl content than the maximum absorption wavebands (i.e. 660–665 nm for Chl *a*) (Zarco-Tejada *et al.*, 2003; Main *et al.*, 2011). Altogether these results suggest a decreasing trend in leaf Chl content under rainfed conditions, with its level of significance depending on the sensitivity of the detection of the spectral parameter (i.e. SPAD meter, SRIs, PROSPECT model).

### Photoprotection-related spectral signal

Besides leaf Chl, the leaf Car and Anth contents are also usually targeted because of their ecophysiological significance (Close and Beadle, 2003; Ustin *et al.*, 2009). Under stress conditions, Cars function to prevent photooxidation of the reaction centres (Ustin *et al.*, 2009), while Anths have an antioxidant and photoprotective role, besides acting as osmoregulators in plant cells (Chalker-Scott, 1999; Steyn *et al.*, 2002; Gould *et al.*, 2002).

Regarding dorsoventrality effects, all Car- and Anth-related SRIs, as well as those related to the Car:Chl ratio, indicated a higher Car and Anth content and Car:Chl ratio in the abaxial leaf side (Supplementary Table S2), suggesting a prevailing photoprotective role for the abaxial side of the leaf. The reported negative correlation between the Anth-related index, mARI (Gitelson *et al.*, 2001), and the epidermis cell sectional area (Fig. 7g) suggests the existence of an anatomy-driven dorsoventral gradient in Anth content. In agreement with this, Cartelat *et al.* (2005) reported higher leaf phenolic content, which includes Anth, in the abaxial side of wheat leaves measured with a portable device (Dualex meter). As reported by Shi *et al.* (2014), this might indicate that the upper part of wheat leaves exhibits greater light use efficiency via lower energy dissipation and photoinhibition. Although the Car estimation by PROSPECT performed similarly to the Car-related SRIs, the differences were not significant. This robust but conservative performance of the PROSPECT parameters may be explained by the large range of leaf pigment concentrations (i.e. wide range of species and conditions) used for the development of the PROSPECT model (Feret *et al.*, 2008). Finally, the results in the flavonol-related index (FRI) (Merzlyak *et al.*, 2005) suggested a possible higher flavonol content in the adaxial leaf side (Supplementary Table S2). It is worth pointing out that this index was originally developed for apple fruit, where Car and Chl contents do not interfere as much as in leaves. Flavonols are considered to have a photoprotective role as UV-absorbing compounds (Mazza *et al.*, 2000), so a possible higher concentration in the upper side of the leaf (i.e. the more exposed to sunlight) (Fig. 7f) may indicate an ecophysiological relevance for them as photoprotectors instead of Anth.

Regarding the water regime effect on photoprotective compounds, Car-related indices were unaffected, and Anth-related indices decreased under rainfed conditions, whereas the flavonol-related SRI increased (Supplementary Table S2). The performance of leaf Cars and Anths in response to water stress is quite variable according to the literature (Alexieva *et al.*, 2001; Chakraborty and Pradhan, 2012; Valifard *et al.*, 2012; Hammad and Ali, 2014). Accumulation of flavonols in the leaves has been reported in wheat in response to water stress and especially in drought-tolerant varieties (Ma *et al.*, 2014). In addition to the photoprotective function mentioned before, previous studies have suggested that flavonols act as antioxidants in plants (Hernández *et al.*, 2009), accumulating in different compartments such as the leaf epidermis (Tevini *et al.*, 1991). In this context, the increase in the ratio of the epidermis section area to the leaf section area (in the adaxial compared with the abaxial leaf side and in rainfed compared with irrigation conditions), which correlated positively with FRI (Fig. 7f), suggested an anatomy-mediated increase in leaf flavonols in response to water stress and to high sunlight levels. These results support the implementation of SRIs related to drought tolerance metabolites for plant stress studies and breeding purposes.

### Main ideas and some insights into the scaling effect and yield prediction

Overall, the detailed study of leaf reflectance and several spectral-derived parameters revealed significant differences between the two sides of the leaf, which in turn are highly dependent on water regime, particularly under irrigated conditions. For instance, the significant interactions between dorsoventral and water regime effects in some of the Car:Chl- and Anth-related indices (Fig. 5; Supplementary Table S2) suggest possible side-specific photochemical changes in response to water regime. Previous studies in monocots (Soares *et al.*, 2008; Soares-Cordeiro *et al.*, 2011) have reported that side-specific physiological responses are involved in leaf acclimation to environmental stresses. In our study, the following tentative trend is hypothesized: in well-watered conditions, the two sides of the leaf are more differentiated, with the adaxial part of the leaf being photosynthetically more efficient (having a lower Car:Chl and Anth), larger epidermal and mesophyll cells, and a thicker epidermis. However, water stress induces alterations in structural, pigment, and photoprotective compounds that tend to reduce dorsoventral differences (functioning) of both leaf sides and consequently the spectroradiometrical response of reflected light. To the best of our knowledge, this is the first study describing a leaf-side-specific response to water regime in wheat using a spectroscopic approach.

The classical approaches for multispectral remote sensing evaluation of leaf and vegetation traits have usually been developed using the reflectance spectrum from the adaxial side of the leaves (Jacquemoud and Baret, 1990; Sims and Gamon, 2002; Lu and Lu, 2015). Although the adaxial leaf spectrum appeared to be more representative of the canopy-level data, the prediction of grain yield was clearly enhanced when abaxial rather than adaxial reflectance is employed, and this was irrespective of whether the full-range spectra or single SRIs were used (Table 4). Therefore, this study reveals that even for a species, such as wheat, with isobilateral leaves, spectra are different, and this may affect the assessment of yield.

Apart from constitutive differences, some anatomical traits of the abaxial leaf side (the epidermis thickness and the epidermis cell area) seemed to be less affected by changing water conditions than the adaxial side. This greater structural homogeneity across the abaxial leaf blade surface may provide a less noisy spectral signal and better suitability for the prediction of yield from spectroscopy compared with the adaxial leaf side.

## Supplementary data

Supplementary data are available at *JXB* online.

Fig. S1. Principal component analysis of reflectances at the leaf and canopy levels.

Fig. S2. Scanning electron micrographs of the epicuticular ultrastructure of flag leaves.

Table S1. Information on the spectral parameters used in this study.

Table S2. Table of means and significance of the two-way ANOVA for the spectral parameters.

Table S3. Significance of the two-way ANOVA of spectral indices for a subset of plots.

Supplementary Figures and Tables
