# Supplementary material for: Leaf dorsoventrality as a paramount factor determining spectral performance in field-grown wheat under contrasting water regimes
Source: J Exp Bot. 2018 Mar 31;69(12):3081–94. doi: 10.1093/jxb/ery109 (PMC5972577; doi:10.1093/jxb/ery109)
Supplement: Supplementary Figures and Tables [file ery109_suppl_supplementary_figures_tables.pdf]

Supplementary data

Article title: Leaf Dorsoventrality as a paramount factor determining spectral performance in field-grown wheat under contrasting water regimes

Authors: Omar Vergara-Diaz, Fadia Chairi, Rubén Vicente, Jose A Fernandez Gallego, Maria Teresa Nieto-Taladriz, Nieves Aparicio, Shawn C. Kefauver, José Luís Araus

The following Supplementary data is available for this article:

Figure S1. Principal component analysis of reflectances introduced as variables. Data points represent the sample measurements and the color labels are indicative of the three levels of measure, at the leaf level adaxial and abaxial reflectance are represented as green and red colour respectively and the canopy reflectance is respresented with the blue colour.

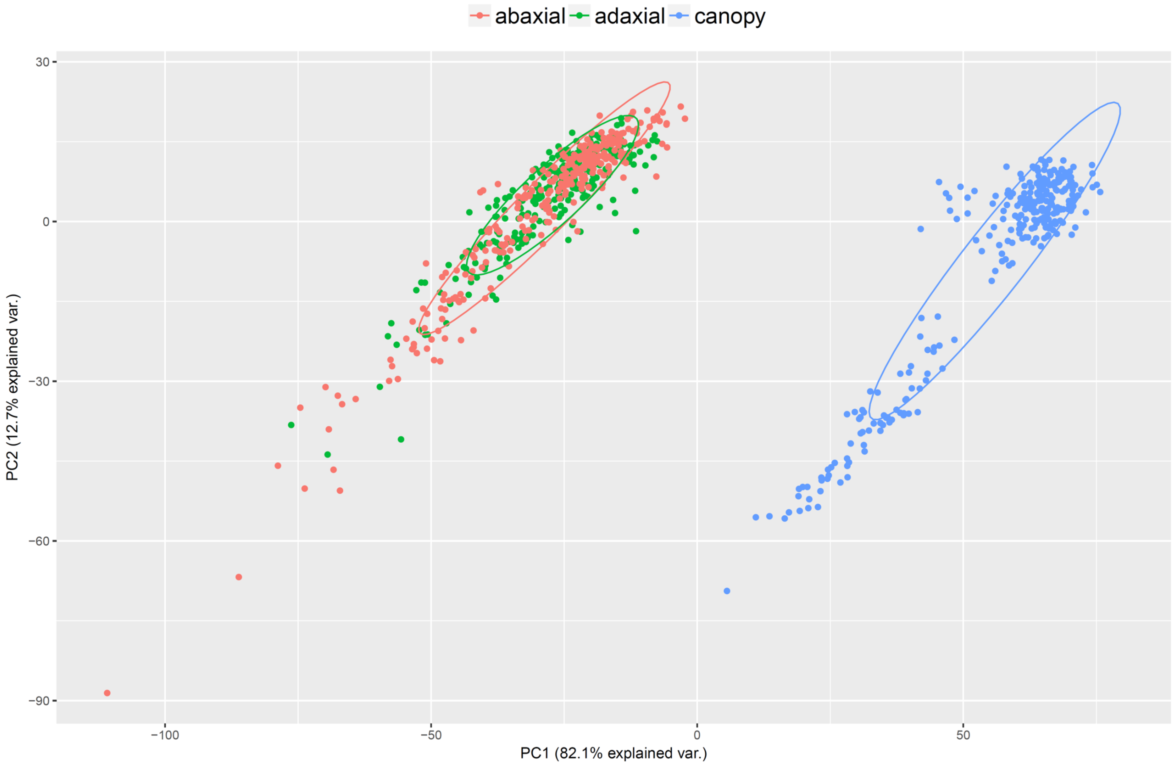

Figure S2. Scanning electron micrographs of the epicuticular ultrastructure of flag leaves. Images were taken from adaxial (A and C) and abaxial (B and D) surfaces of durum wheat grown under supplemental irrigation (A and B) and rainfed conditions (C and D) observed at 10000x magnification.

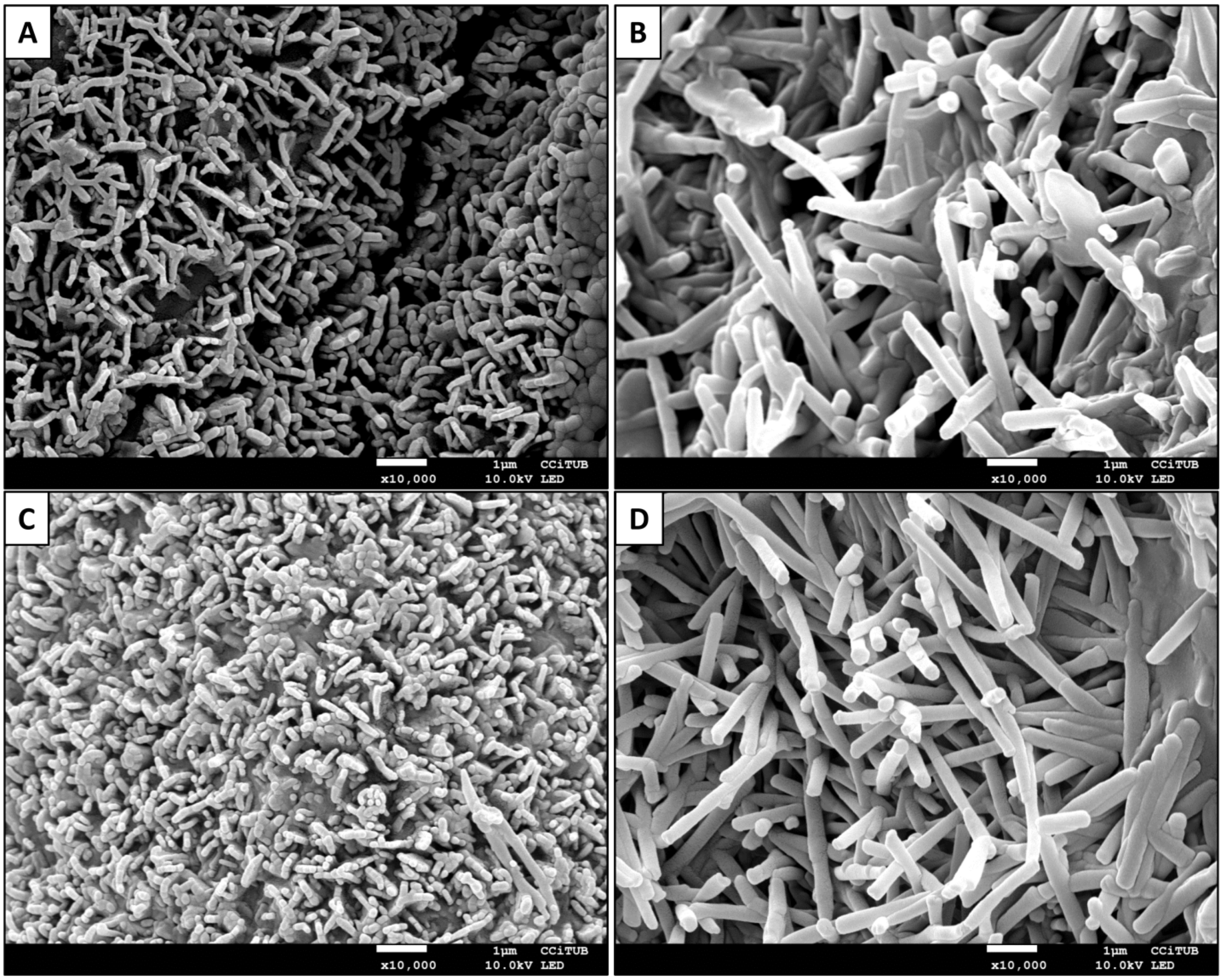

Table S1 Information on the spectral parameters used in this study, including their names and acronyms, target traits, formulations and citing literature. Subscripts refer to the wavelength used in the calculations of the spectral reflectance indices.

Index	Name	Target trait(s)	Formula	Literature
MSI	Moisture stress index	Water content	R_{1599} / R_{819}	Hunt & Rock, 1989
NDII	Normalised difference infrared index	Water content	$(R_{819} - R_{1649})/(R_{819} + R_{1649})$	Hardisky et al. 1983
NWI_1	Normalised difference water index 1	Water content	$(R_{970} - R_{900})/(R_{970} + R_{900})$	Babar et al. 2006
NWI_2	Normalised difference water index 2	Water content	$(R_{970} - R_{920})/(R_{970} + R_{920})$	Prasad et al. 2007
NWI_3	Normalised difference water index 3	Water content	$(R_{970} - R_{880})/(R_{970} + R_{880})$	Gao et al. 1995
NDWI	Normalised difference water index	Canopy water content	$(R_{857}-R_{1241})/(R_{857}+R_{1241})$	Gao 1996
NMDI	Normalised multi-band drought index	Drought status	$(R_{860}-(R_{1640} - R_{2130}))/(R_{860} + (R_{1640} - R_{2130}))$	Wang & Qu, 2007
WBI	Water band index	Canopy water status	R_{970} / R_{900}	Peñuelas et al. 1993
WI	Water index	Canopy water status	R_{900} / R_{970}	Peñuelas et al. 1993
$NDMI_1$	Normalised difference moisture index 1	Vegetation water content	$(R_{2200} - R_{1100})/(R_{2200} + R_{1100})$	Wilson & Sader, 2002
$NDMI_2$	Normalised difference moisture index 2	Moisture	$(R_{2247} - R_{1147})/(R_{2247} + R_{1147})$	Lobos et al. 2014
$NDMI_3$	Normalised difference moisture index 3	Moisture	$(R_{1650} - R_{830})/(R_{1650} - R_{850})$	Inoue et al. 2007
SWWI	Short Wave Water Index	Water content	(R_{1650} / R_{850})	Lobos et al. 2014
RGRR	Red green reflectance ratio	Anthocyanin content	R_{Red}/R_{Green}	Gamon & Surfus 1999
ARI	Anthocyanin reflectance index	Anthocyanin content	$R_{550}^{-1} - R_{700}^{-1}$	Gitelson et al. 2001
mARI	Modified anthocyanin reflectance index 1	Anthocyanin content	$R_{800} \times (R_{550}^{-1} - R_{700}^{-1})$	Gitelson et al. 2001
GATB	Gitelson Anthocyanin three	Anthocyanin content	$(R_{553-570}^{-1} - R_{690-710}^{-1}) \times R_{760-800}$	Gitelson et al. 2006

	band			
CRI_1	Carotenoid reflectance index 1	Carotenoid content	$(1/R_{510})$ - $(1/R_{550})$	Gitelson et al. 2002
CRI_2	Carotenoid reflectance index 2	Carotenoid content	$(1/R_{510})$ - $(1/R_{700})$	Gitelson et al. 2002
CRI_3	Carotenoid reflectance index 3	Carotenoid content	R_{800} x $((1/R_{520})-(1/R_{550}))$	Getilson et al. 2002
RARS-Car	Ratio analysis of reflectance spectra-Car	Carotenoid content	R_{746}/R_{513}	Chappelle et al. 1992
$PSSR_c$	Pigment-specific simple ratio-Car	Carotenoid content	R_{800}/R_{470}	Blackburn, 1998
ChlNDI	Chlorophyll normalised difference index	Chlorophyll a content	$(R_{705} - R_{445})/(R_{750} + R_{445})$	Datt, 1999
RECI	Red-edge Chlorophyll Index	Chlorophyll content	$(R_{770-800}/R_{720-730}) – 1$	Gitelson et al. 2003
mDATT	Modified Datt index	Chlorophyll content	$(R_{719} – R_{726})/(R_{719} – R_{743})$	Lu et al. 2015
TCI	Triangular chlorophyll index	Chlorophyll content	$1.2(R_{700}-R_{550})-1.5(R_{670}-R_{550})$ x $(R_{700}/R_{670})^{0.5}$	Haboudane et al. 2008
TCARI	Transformed chlorophyll absorption reflectance index	Chlorophyll content	3x$((R_{700}-R_{670})-0.2$ x $(R_{700}-R_{550})$ x $(R_{700}/R_{670}))$	Rondeaux et al. 1996
NPQI	Normalized Phaeophytinization Index	Chlorophyll degradation	$(R_{415}-R_{435})(R_{414}+R_{435})$	Barnes et al. 1992
mCARI	Modified chlorophyll absorption reflectance index	Chlorophyll content	$(1.5$ x $(R_{800}-R_{670})$ - 1.3 x $(R_{800} -R_{550}))$/ √ ((2 x R_{800} + 1) x (2 x R_{800} +1) - (6 x R_{800} - 5 x $\sqrt{R_{670}}$) - 0.5)	Haboudane et al. 2004
CI	Carter index	Plant stress	R_{760} / R_{695}	Carter, 1994
$VREI_1$	Vogelmann red edge index 1	Chlorophyll content	(R_{740} / R_{720})	Vogelmann et al. 1993
$VREI_2$	Vogelmann red edge index 2	Chlorophyll content	$(R_{734} - R_{747})/(R_{715} + R_{726})$	Vogelmann et al. 1993
RENDVI	Red-Edge normalized difference index	Chlorophyll content	$(R_{750} - R_{705})/(R_{750} + R_{705})$	Gitelson & Merzlyak 1994
mRENDVI	Modified normalized difference index	Chlorophyll content	$(R_{750} - R_{705})/(R_{750} + R_{705} - 2$ x $R_{445})$	Sims & Gamon 2002
MRCI	MERIS total chlorophyll index	Chlorophyll content	$(R_{750}-R_{710})/(R_{710}-R_{680})$	Dash & Curran 2004
$PSSR_a$	Pigment-specific simple ratio	Chl a content	R_{800}/R_{680}	Blackburn, 1998

$PSSR_b$	Pigment-specific simple ratio	Chl b content	R_{800}/R_{635}	Blackburn, 1998
NDRE	Normalised difference red edge	Chlorophyll content	$(R_{790}-R_{720})/(R_{790}+R_{720})$	Barnes et al. 2000
PSRI	Plant senescing reflection index	Carotenoid to chlorophyll ratio	$(R_{680}-R_{500})1/R_{750}$	Merzlyak et al. 1999
SIPI	Structural independent pigment index	Carotenoid to chlorophyll a ratio	$(R_{800} - R_{445})/(R_{800} - R_{680})$	Peñuelas et al 1995;
PRI	Photochemical reflectance index	Photosynthetic light-use efficiency , Car/Chl ratio	$(R_{531} - R_{570})/(R_{531} + R_{570})$	Gamon et al. 1992
NPCI	Normalised pigment chlorophyll ratio index	Car/Chl a ratio; senescense, N status	$(R_{680}-R_{430})/(R_{430}+R_{680})$	Peñuelas et al. 1993
SRPI	Simple ratio pigment index	Car/Chl a ratio	R_{430}/R_{680}	Peñuelas et al. 1995
FRI	Flavonols reflectance index	Flavonol content	$(R_{410}^{-1} - R_{460}^{-1}) \times R_{800}$	Merzylak et al. 2005
NDNI	Normalised difference nitrogen index	Nitrogen content	$(\log(R_{1510}^{-1}) - \log(R_{1680}^{-1})) / (\log(R_{1510}^{-1}) + \log(R_{1680}^{-1}))$	Serrano et al 2002
NDLI	Normalized difference lignin index	Lignin content	$(\log(R_{1754}^{-1}) - \log(R_{1680}^{-1}))/(\log(R_{1754}^{-1})+\log(R_{1680}^{-1}))$	Serrano et al. 2002
CAI	Cellulose absorption index	Dried plant material and cellulose	$0.5 \times (R_{2000} + R_{2200}) - R_{2100}$	Nagler et al. 2000
NDryMI	Normalized dry matter index	Dry matter content and water status	$(R_{1649}-R_{1722})/(R_{1649}+R_{1722})$	Hunt et al. 2012
$NDVI_1$	Normalised difference vegetation index	Green biomass	$(R_{800} - R_{670})/(R_{800} + R_{670})$	Rouse et al. 1974
GVI	Greenness Vegetation index	Green biomass	$(R_{682} - R_{553})/(R_{682} + R_{553})$	Not found
$NDVI_w$	Normalised difference vegetation index	Green biomass	$(R_{830} - R_{660})/(R_{830} + R_{660})$	Rouse et al. 1973
$NDVI_n$	Normalized difference vegetation index	Green biomass	$(R_{760} - R_{660})/(R_{760} + R_{660})$	Rouse et al. 1973
$GNDVI_n$	Green normalized difference vegetation index	Green biomass	$(R_{870} - R_{568})/(R_{870} + R_{568})$	Gitelson et al. 1996
$GNDVI_w$	Green normalized difference vegetation index	Green biomass	$(R_{830} - R_{560})/(R_{830} + R_{560})$	Gitelson et al. 1996

GNDVI	Green normalized difference vegetation index	Green biomass	$(R_{800}-R_{550})/(R_{800}+R_{550})$	Daughtry et al. 2000
SR_1	Simple ratio	Green biomass	R_{900} / R_{680}	Rouse et al. 1973
SR_2	Simple ratio	Green biomass	R_{770} / R_{680}	Not found
SR_3	Simple ratio	Chlorophyll content	R_{750} / R_{700}	Gitelson & Merzlyak 1997
SR_4	Simple ratio 4	Chlorophyll content	R_{850} / R_{710}	Datt, 1999
mSR_1	Modified simple ratio 1	Chlorophyll content	$(R_{850} - R_{710})/(R_{850} - R_{680})$	Datt, 1999
mSR_2	Modified simple ratio 2	Chlorophyll content	$(R_{780} - R_{710})/(R_{780} - R_{680})$	Maccioni et al. 2001
mSR_3	Modified simple ratio 3	Chlorophyll content	$(R_{750} - R_{445})/(R_{705} - R_{445})$	Sims & Gamon 2002
mSR_4	Modified simple ratio 4	Leaf area index and biomass	$(R_{800}/R_{670}-1)/(\sqrt{} (R_{800}/R_{670}+1))$	Chen 1996
TVI	Triangular vegetation index	Leaf area index and Chlorophyll content	$0.5 \times (120 \times (R_{750}-R_{550}) - 200 \times (R_{670}-R_{550})$	Broge & Leblanc 2000
$mTVI_1$	Modified triangular vegetation index 1	Leaf area index	$1.2\ (1.2 \times (R_{800}-R_{550}) – 2.5 \times (R_{670}-R_{550})$	Haboudane et al. 2004
$mTVI_2$	Modified triangular vegetation index 2	Leaf area index	$(1.5 \times (1.2 \times (R_{800}-R_{550}) - 2.5 \times (R_{670}-R_{800})))/ \sqrt{} ((2 \times R_{800} + 1) \times (2 \times R_{800} + 1)-(6 \times R_{800} - 5 \times \sqrt{R_{670}} - 0.5))$	Haboudane et al. 2004
OSAVI	Optimized soil-adjusted vegetation index	Vegetation cover and biomass	$(1.5 \times (R_{800}-R_{670})/(R_{800}+R_{670}+0.16)$	Rondeaux et al. 1996
GI	Greeness index	Greenness	R_{554}/R_{677}	Zarco-Tejada et al. 2005
EVI_2	Enhanced vegetation index 2	Green biomass	$2.5 \times (R_{800}-R_{660})/(1+R_{800}+2.4*R_{660})$	Jiang et al. 2008
RDVI	Renormalised difference vegetation index	Green biomass	$(R_{800}-R_{670})/(\sqrt{R_{800}}+R_{670})$	Rougean & Breon 1995
TDVI	Transformed difference vegetation index	Vegetation cover and biomass	$\sqrt{} (0.5 +((R_{NIR}-R_{Red})/(R_{NIR}+R_{Red})))$	Deering et al. 1975
RVI	Ratio vegetation index	Vegetation cover and health	R_{NIR} / R_{Red}	Birth et al. 1968
OSAVI	Optimized soil-adjusted vegetation index	Vegetation cover and biomass	$(1.5*(R_{NIR} - R_{Red}))/(R_{NIR}+R_{red} + 0.16)$	Rondeaux et al. 1996
NDVI	Normalised difference vegetation index	Vegetation cover and biomass	$(R_{NIR} - R_{Red})/(R_{NIR} + R_{Red})$	Rouse et al. 1973

NLI	Non-linear index	Vegetation cover and biomass	$(R_{NIR}^2 - R_{Red})/(R_{NIR}^2 + R_{Red})$	Goel & Qin, 1994
mNLI	Modified non-linear index	Vegetation cover and biomass	$((R_{NIR}^2 - R_{Red}) \times 1.5)/((R_{NIR}^2 + R_{Red}) + 0.5)$	Yang et al. 2008
IPVI	Infrared percentage vegetation index	Vegetation cover and biomass	$R_{NIR}/(R_{NIR} + R_{Red})$	Crippen, 1990
GRVI	Green ratio vegetation index	Canopy cover	R_{NIR}/R_G	Sripada et al. 2006
EVI	Enhanced vegetation index	Canopy cover	$2.5 \times ((R_{NIR}-R_{Red})/(R_{NIR}+ 6 \times R_{Red}-7.5 \times R_B+1))$	Liu & Huete 1995
GNDVI	Green normalised difference vegetation index	Green biomass	$(R_{NIR} - R_G)/(R_{NIR} + R_G)$	Gitelson & Merzlyak 1998
RNDVI	Red normalised difference vegetation index	Healthy vegetation	$(R_{NIR-R})/ \sqrt{(R_{NIR}+R_{Red})}$	Roujean & Breon 1995
TCARI/OSAVI	Index Ratio	Chlorophyll content	TCARI/OSAVI	Haboudane et al. 2002
WI/NDVI	Index Ratio	Plant water content	WBI/NDVI	Peñuelas et al 1997
PROSPECT5 model				
Cab	Chlorophyll content			Jacquemoud & Barert 1990
Cxc	Carotenoid content			
EWT	Equivalent water thickness			

Table S2 Mean values of the spectral parameters for each water regime (R+, irrigated; R-, rainfed) and in each side of the leaf, together with the significance levels of the two-way analysis of variance**.**

Target trait	Water Regime		Leaf Side		Adaxial		Abaxial		Significance		
	R+	R-	adaxial	abaxial	R+	R-	R+	R-	P_{WR}	P_{LS}	P_{WRxLS}
Water content											
MSI	0.559	0.573	0.574	0.559	0.567	0.580	0.552	0.566	0.000	0.000	0.907
NDII	0.237	0.227	0.227	0.238	0.232	0.222	0.243	0.232	0.000	0.000	0.790
NWI_1	-0.016	-0.015	-0.016	-0.016	-0.016	-0.016	-0.016	-0.015	0.249	0.301	0.712
NWI_2	-0.016	-0.016	-0.016	-0.016	-0.016	-0.016	-0.016	-0.015	0.052	0.280	0.659
NWI_3	-0.015	-0.015	-0.015	-0.015	-0.015	-0.015	-0.015	-0.014	0.406	0.391	0.767
NDWI	0.042	0.037	0.039	0.040	0.041	0.037	0.043	0.038	0.000	0.007	0.845
NMDI	0.459	0.453	0.450	0.462	0.452	0.447	0.465	0.458	0.000	0.000	0.420
WBI	0.969	0.970	0.969	0.969	0.968	0.969	0.969	0.970	0.249	0.302	0.714
WI_2	1.032	1.032	1.032	1.032	1.033	1.032	1.032	1.031	0.249	0.299	0.711
$NDMI_1$	-0.578	-0.568	-0.572	-0.574	-0.577	-0.568	-0.579	-0.569	0.002	0.676	0.786
$NDMI_2$	-0.575	-0.568	-0.571	-0.572	-0.574	-0.568	-0.576	-0.568	0.016	0.804	0.826
$NDMI_3$	0.986	0.985	0.985	.987	0.986	0.984	0.987	0.987	0.010	0.000	0.135
SWWI	0.613	0.626	0.626	0.613	0.620	0.633	0.606	0.620	0.000	0.000	0.784
Anthocyanin											
ARI	-0.328	-0.452	-0.635	-0.144	-0.627	-0.644	-0.029	-0.259	0.000	0.000	0.001
mARI	-0.281	-0.387	-0.544	-0.124	-0.537	-0.551	-0.026	-0.222	0.000	0.000	0.001
RGRR	0.685	0.676	0.673	0.688	0.677	0.669	0.692	0.683	0.126	0.010	0.950
GATB	0.931	0.817	0.688	1.060	0.670	0.706	1.193	0.927	0.006	0.000	0.000
Carotenoids											
CRI_1	6.058	6.146	5.707	6.496	5.513	5.902	6.603	6.389	0.681	0.000	0.157
CRI_2	5.730	5.694	5.072	6.352	4.886	5.258	6.574	6.130	0.865	0.000	0.056
CRI_3	2.871	2.883	2.773	2.982	2.707	2.839	3.036	2.927	0.895	0.017	0.168
RARS-Car	10.370	10.275	9.978	10.667	9.834	10.122	10.907	10.428	0.646	0.001	0.066
$PSSR_c$	13.398	13.413	12.641	14.170	12.349	12.932	14.446	13.893	0.965	0.000	0.091
Chlorophyll											
ChlNDI	0.113	0.116	0.114	0.115	0.111	0.116	0.115	0.116	0.178	0.523	0.411
RECI	0.852	0.825	0.819	0.858	0.836	0.803	0.867	0.847	0.012	0.000	0.586
mDATT	0.324	0.329	0.332	0.322	0.329	0.335	0.320	0.323	0.000	0.000	0.213
mSR_1	0.792	0.785	0.785	0.792	0.788	0.782	0.795	0.789	0.038	0.023	0.921
mSR_2	0.788	0.782	0.781	0.788	0.785	0.778	0.791	0.785	0.035	0.020	0.915
mSR_3	7.826	8.112	7.767	8.171	7.905	7.629	7.747	8.596	0.576	0.431	0.273
TCARI	0.185	0.191	0.191	0.185	0.189	0.193	0.180	0.190	0.096	0.135	0.547
mCARI	0.134	0.138	0.133	0.139	0.130	0.136	0.138	0.140	0.444	0.243	0.773
CI	10.286	10.267	10.326	10.228	10.242	10.409	10.331	10.124	0.915	0.597	0.315
$VREI_1$	1.852	1.826	1.822	1.856	1.836	1.809	1.868	1.844	0.009	0.001	0.897
$VREI_2$	-0.199	-0.191	-0.189	-0.201	-0.194	-0.184	-0.205	-0.198	0.006	0.000	0.678
RENDVI	0.646	0.643	0.646	0.643	0.647	0.646	0.645	0.640	0.372	0.346	0.576
mRENDVI	0.764	0.758	0.762	0.760	0.766	0.758	0.761	0.759	0.217	0.696	0.522
MRCI	3.424	3.300	3.304	3.420	3.365	3.243	3.482	3.358	0.014	0.021	0.980
NDRE	0.388	0.381	0.379	0.390	0.384	0.375	0.393	0.387	0.021	0.002	0.683
$PSSR^a$	15.238	15.297	15.049	15.486	14.861	15.237	15.615	15.356	0.871	0.224	0.377
$PSSR^b$	13.580	13.340	13.054	13.866	12.915	13.193	14.245	13.488	0.428	0.007	0.087

NPQI	-0.002	-0.008	-0.021	0.011	-0.018	-0.024	0.013	0.008	0.000	0.000	0.661
Car/Chl ratio											
PSRI	-0.010	-0.010	-0.012	-0.008	-0.013	-0.012	-0.006	-0.009	0.069	0.000	0.000
SIPI	0.987	0.986	0.984	0.988	0.983	0.985	0.990	0.987	0.524	0.000	0.002
PRI	0.039	0.037	0.035	0.041	0.035	0.035	0.043	0.038	0.069	0.000	0.061
NPCI	-0.085	-0.082	-0.094	-0.073	-0.101	-0.086	-0.069	-0.078	0.464	0.000	0.001
SRPI	1.191	1.185	1.212	1.163	1.229	1.195	1.152	1.175	0.534	0.000	0.001
Other											
FRI	-0.337	-0.042	0.551	-0.930	0.402	0.701	-1.075	-0.784	0.000	0.000	0.955
TCARI/OSAVI	0.166	0.172	0.171	0.167	0.170	0.173	0.163	0.171	0.089	0.209	0.461
WBI/NDVI	1.118	1.117	1.111	1.124	1.113	1.108	1.123	1.125	0.842	0.024	0.572
Nitrogen content											
NDNI	0.311	0.306	0.310	0.307	0.313	0.307	0.310	0.304	0.000	0.027	0.939
Structural C											
NDLI	0.109	0.110	0.112	0.107	0.111	0.113	0.106	0.107	0.000	0.000	0.240
CAI	-0.017	-0.017	-0.019	-0.016	-0.019	-0.019	-0.016	-0.016	0.752	0.000	0.627
NDryMI	0.037	0.039	0.039	0.036	0.038	0.040	0.036	0.037	0.000	0.000	0.577
Narrow-band greenness											
$NDVI_1$	0.870	0.871	0.873	0.867	0.872	0.875	0.868	0.867	0.722	0.098	0.534
GVI	-0.338	-0.353	-0.357	-0.335	-0.351	-0.362	-0.325	-0.344	0.050	0.003	0.582
$NDVI_w$	0.869	0.869	0.871	0.867	0.870	0.873	0.868	0.866	0.855	0.228	0.417
$NDVI_n$	0.864	0.865	0.867	0.862	0.865	0.869	0.863	0.861	0.855	0.240	0.403
$GNDVI_n$	0.781	0.776	0.777	0.780	0.776	0.777	0.785	0.775	0.235	0.333	0.150
$GNDVI_w$	0.758	0.753	0.753	0.758	0.753	0.753	0.763	0.753	0.176	0.224	0.153
GNDVI	0.748	0.743	0.743	0.747	0.743	0.743	0.753	0.742	0.181	0.225	0.157
SR_1	15.527	15.588	15.344	15.771	15.149	15.540	15.905	15.637	0.867	0.243	0.368
SR_2	15.033	15.091	14.845	15.280	14.657	15.033	15.410	15.150	0.870	0.220	0.369
SR_3	6.81	6.78	6.867	6.724	6.839	6.895	6.788	6.660	0.716	0.150	0.355
SR_4	3.83	3.77	3.785	3.814	3.813	3.757	3.849	3.779	0.124	0.485	0.864
TVI	52.85	53.17	53.42	52.61	53.26	53.57	52.45	52.77	0.251	0.003	0.980
mTVI1	1.255	1.255	1.259	1.250	1.258	1.260	1.251	1.250	0.979	0.051	0.684
mTVI2	2.525	2.523	2.525	2.522	2.520	2.531	2.530	2.515	0.858	0.815	0.315
OSAVI	1.111	1.112	1.115	1.108	1.113	1.117	1.109	1.107	0.871	0.126	0.471
GI	2.148	2.212	2.211	2.150	2.184	2.237	2.113	2.187	0.915	0.597	0.315
EVI2	0.997	0.996	0.998	.995	0.996	1.000	0.998	0.991	0.671	0.502	0.264
RDVI	0.835	0.834	0.836	.833	0.835	0.836	0.835	0.831	0.678	0.293	0.328
Broad-band greenness											
TDVI	1.167	1.167	1.168	1.166	1.168	1.169	1.167	1.166	0.879	0.195	0.442
RVI	14.90	14.77	14.45	15.22	14.27	14.63	15.52	14.92	0.724	0.027	0.162
mSR_4	2.815	2.806	2.781	2.841	2.756	2.805	2.875	2.807	0.841	0.199	0.214
OSAVI	1.103	1.103	1.105	1.100	1.103	1.107	1.102	1.098	0.965	0.275	0.370
NDVI	0.862	0.863	0.865	0.860	0.863	0.867	0.862	0.859	0.885	0.228	0.432
NLI	0.843	0.843	0.844	0.841	0.843	0.846	0.843	0.839	0.998	0.363	0.382
mNLI	1.764	1.764	1.767	1.761	1.764	1.769	1.764	1.758	0.998	0.363	0.382
IPVI	0.931	0.932	0.933	0.930	0.932	0.933	0.931	0.930	0.885	0.228	0.432
GRVI	8.611	8.436	8.340	8.706	8.302	8.378	8.919	8.494	0.231	0.012	0.086
EVI	1.169	1.166	1.172	1.163	1.175	1.169	1.163	1.163	0.351	0.003	0.368
GNDVI	0.784	0.780	0.781	0.784	0.780	0.781	0.788	0.779	0.278	0.424	0.164
RNDVI	0.830	0.828	0.830	0.828	0.829	0.831	0.831	0.825	0.546	0.517	0.239
PROSPECT											
Cab	52.9	52.28	52.35	52.84	52.47	52.22	53.34	52.34	0.481	0.578	0.669
Cxc	13.64	13.84	13.54	13.93	13.15	13.94	14.12	13.74	0.627	0.353	0.164
EWT	0.013	0.012	0.012	0.012	0.013	0.012	0.013	0.012	0.001	0.968	0.846

Table S3 Significance levels of the two-way analysis of variance for a selection of spectral reflectance indices depending on water regime and on the side of the leaf in the subset of plots selected for the anatomical measurements.

	P_{WR}	P_{LS}	P_{WR*LS}
PRI	0.168	0.029	0.121
ARI	0.026	< 0.001	0.005
mARI	0.038	< 0.001	0.005
NDLI	0.540	< 0.001	0.961
CAI	0.156	0.012	0.802
MSI	0.515	0.371	0.872
PSRI	0.061	0.025	< 0.001
NDWI	0.014	0.219	0.853
NMDI	0.112	0.005	0.778
NDNI	< 0.001	0.347	0.924
MDATT	0.128	0.027	0.597
$NDMI_3$	0.248	0.020	0.792
Chl – RM	0.003	0.011	0.425
NDryMI	< 0.001	0.008	0.752
